# Systematic review of gut microbiota and attention-deficit hyperactivity disorder (ADHD)

**DOI:** 10.1186/s12991-021-00330-w

**Published:** 2021-02-16

**Authors:** Alverina Cynthia Sukmajaya, Maria Inge Lusida, Soetjipto, Yunias Setiawati

**Affiliations:** 1 Faculty of Medicine, Universitas Airlangga, Surabaya, Indonesia; 2 Institute of Tropical Disease, Universitas Airlangga, Surabaya, Indonesia; 3 Department of Psychiatric, Faculty of Medicine Universitas Airlangga, Dr. Soetomo General Hospital, Surabaya, Indonesia; 4 Department of Microbiology, Faculty of Medicine, Universitas Airlangga, Surabaya, Indonesia

**Keywords:** Attention-deficit hyperactivity disorder (ADHD), Gut, Microbiota, Gut–brain axis (GBA)

## Abstract

**Background:**

Gut–brain axis (GBA) is a system widely studied nowadays, especially in the neuropsychiatry field. It is postulated to correlate with many psychiatric conditions, one of them being attention-deficit hyperactivity disorder (ADHD). ADHD is a disorder that affects many aspects of life, including but not limited to financial, psychosocial, and cultural aspects. Multiple studies have made a comparison of the gut microbiota between ADHD and healthy controls. Our aims were to review the existing studies analyzing the gut microbiota between human samples in ADHD and healthy individuals.

**Methods:**

The literature was obtained using Google Scholar, Pubmed, and Science Direct search engine. The keywords used were “ADHD”, “gut microbiota”, “stool”, “gut”, and “microbiota”. The selected studies were all case–control studies, which identify the gut microbiota between ADHD and healthy individuals.

**Result:**

We found six studies which were eligible for review. The model and methods of each study is different. Forty-nine bacterial taxa were found, yet none of them can explain the precise relationship between ADHD and the gut microbiota. *Bifidobacterium* was found in higher amount in ADHD patients, but other study stated that the abundance of this genus was lower in ADHD with post-micronutrient treatment. This may suggest that micronutrient can modulate the population of *Bifidobacterium* and improve the behavior of ADHD patients*.* Other notable findings include a significantly lower population of *Dialister* in unmedicated ADHD, which rose after patients were medicated. A smaller amount of *Faecalibacterium* were also found in ADHD patients. This may explain the pathogenesis of ADHD, as *Faecalibacterium* is known for its anti-inflammatory products. It is possible the scarcity of this genera could induce overproduction of pro-inflammatory cytokines, which is in accordance with the high level of pro-inflammatory cytokines found in children with ADHD.

**Conclusion:**

There were no studies that examined which bacterial taxa correlated most to ADHD. This might occur due to the different model and methods in each study. Further study is needed to identify the correlation between gut microbiota and ADHD.

## Introduction

Attention-deficit hyperactivity disorder (ADHD) is a neurodevelopmental disorder affecting 7.2% children below 18 years old globally [1]. The prevalence keeps increasing for the last 20 years [2]. Although people with ADHD generally have good quality of life, some of them may experience difficulties in navigating daily life. Without proper treatment, ADHD may lead to some serious consequences, such as academic failure, social disruption, unwanted accidents, strained family relationships, and disorganized career. Furthermore, ADHD is a disorder that affects many aspects of life, including financial, psychosocial, and cultural aspects. The financial burden of ADHD ranges from $503 to $1.343 annually, which comprises hospitalization expenses, psychiatry consultations, and medicines [3]. In addition, it is reported that parents tend to resign from their jobs to provide more attention to their ADHD children. Children with ADHD also need extra help from their teachers at school, but such assistance is not always optimally provided due to the workload of said teachers [4].

Gut–brain axis (GBA) is a system widely studied right now because of the novel understanding that the gut microbiota environment can affect brain activity and vice versa. The bidirectional communication involves the central nervous system, brain and spinal cord, autonomic nervous system, enteric nervous system, and hypothalamic pituitary adrenal (HPA) axis [5]. Unbalanced microbiota composition, known as dysbiosis, is caused by the increase in inflammatory microbes that may impair gut permeability. This in turn can cause microbial translocation, which leads to systemic inflammation. A systemic inflammation may trigger the disruption of blood–brain barrier and increase the level of pro-inflammatory cytokines such as IL-6 and IFN-γ. In addition, dysbiosis may produce oxidative stress that affects neuron cell and neurotransmitters related to ADHD [6].

A study conducted in animal models has found that several taxa differed significantly between mice that were colonized by ADHD microbiota and healthy control mice. Fourteen genera increased, while 17 genera were found to be more abundant in control. Mice with transplanted microbiota displayed abnormalities in their brain such as decreased integrity in both white and gray matter regions. Other than that, the MRI result showed a decreased resting-state connectivity between right motor and visual cortices [7].

Finally, several studies have compared the gut microbiota in ADHD with healthy individuals. We systematically reviewed the scientific literature of case–control studies focusing on gut microbiota composition in ADHD.

## Methods

### Literature search for gut microbiota studies in ADHD

The population of this systematic review is ADHD patient, the intervention is the profiles of gut microbiota, the comparator is healthy control, and the outcome of this study is to find any differences in gut microbiota profiles between ADHD and healthy controls. The literature search was conducted using Google Scholar, PubMed, and Science Direct with keywords “ADHD”, “gut microbiota”, “stool”, “gut”, and “microbiota”. The studies were then selected based on the inclusion and exclusion criterias. The inclusion criteria include: (1) all studies must be in English and discuss gut microbiota in ADHD; (2) the samples in the studies were diagnosed based on Diagnostic and Statistical Manual of Mental Disorders (DSM) 4th or 5th edition or ICD-10 with the code F90.9; (3) the studies conducted in human and the gut microbiota were identified using fecal samples. We excluded studies with samples below ten person(s) and studies that focus on other condition besides ADHD, such as interventional study.

## Results

### Literature search

**Fig. 1:**
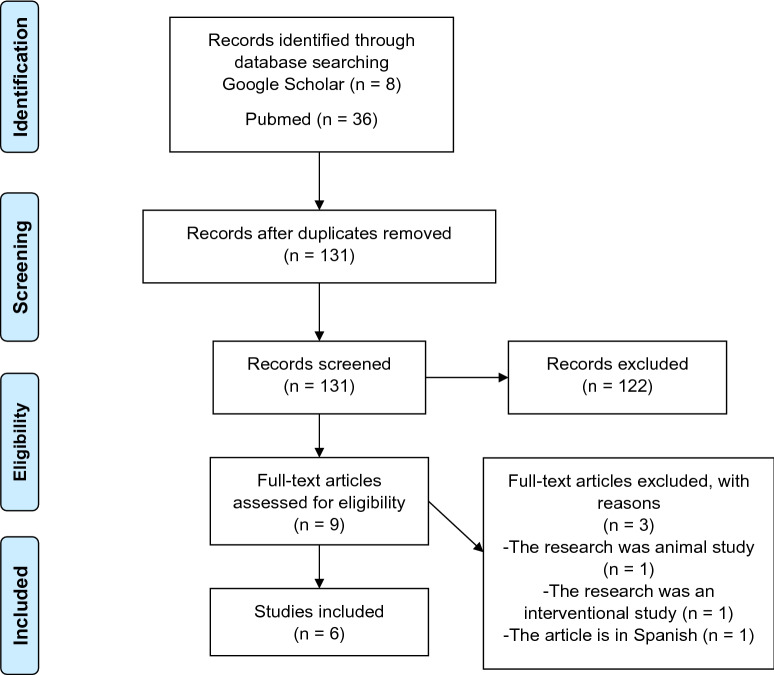
PRISMA

The studies selection was conducted using Preferred Reporting Items for Systematic Reviews and Meta-Analyses (PRISMA) [8]. From the database search, we found 146 studies with the keywords. We removed duplication and found 131 studies. After screening through the titles and abstracts, we found 122 potential studies to be reviewed. From that number we narrowed it down to 9 eligible studies to be reviewed, but 3 must be removed (one was an animal study, one was an interventional study, and another was in Spanish). Finally, we found six case–control studies ranging from 2017 until 2020, involving 407 research participants, 172 ADHD, 15 subthreshold ADHD, and 220 healthy controls (see Fig. 1).

### Characteristics of studies (see Table ***2******)***

#### Samples in studies

Three out of six studies were conducted in Asia [9–11] (Beijing, Taiwan, and Zhejiang). Two other studies were conducted in the Netherlands [12,13] and the other one was conducted in Germany [14]. The samples for each study varied between 14 and 77 participants.

#### Dietary pattern

Two studies [12,13] did not explain about the dietary pattern in their samples, but the healthy controls from study by Aarts et al. were selected from the healthy siblings of the ADHD patients. Therefore, the comparison between gut microbiome in ADHD patients and healthy controls might be more accurate, as dietary variations can be eliminated from the factors influencing gut microbiome, further homogenizing samples. One study [14] recorded the dietary pattern only for fast food, meat/sausage/cold cuts, fruits/vegetables, and yoghurt/dairy products. Two studies used questionnaires for their samples’ dietary log; one used a questionnaire filled by patients’ parents [9] and the other one used Food Frequency Questionnaire (FFQ) [10]. The last study [11] stated that their samples were asked to maintain their regular dietary pattern for 1 week and record their food intake using food diary.

#### ADHD medication and consumption of probiotic/prebiotic/antibiotic

Out of all studies, only two studies [13,14] included individuals with ADHD medication. Two other studies [9,10] excluded those who have a history of ADHD medication, while the rest [11, 12] did not mention this criteria in their articles. Other than that, only one study [9] excluded prebiotic/antibiotic use within 2 months prior to samples collection. One study [10] also excluded prebiotic/antibiotic use, but did not explain the duration of consumption. On the other hand, one study [11] specifically excluded the use of probiotic within 1 month prior to samples collection. Finally, the last study [13] stated that they could not evaluate the use of probiotic/prebiotic/antibiotic in their samples.

#### Psychiatric condition, allergic history, and other medical conditions

Two studies [9, 11] excluded depressive and anxiety symptoms in their samples along with atopic history (asthma, eczema, and allergic rhinitis). In addition, they also excluded samples with a history of digestive or chronic diseases. Only one study [10] stated that they excluded all neuropsychiatric conditions except ADHD and other major diseases. The other studies did not give any information about this criterion. Other than that, the study by Jiang et al. [9] excluded samples with obesity and Wan et al. [11] excluded samples with body mass index (BMI) below 20 kg/m^2^.

### Methodology

#### Clinical assessments

The diagnostic tools included K-SADS (Kiddie Schedule for Affective Disorders and Schizophrenia) based on DSM-IV, DSM-IV-TR, and DSM-5. Three studies [11–13] used K-SADS to diagnose their samples. Two other studies [9, 14] used K-SADS-PL, the present and lifetime version which is used for affective and psychotic disorders screening, as well as other disorders, such as major depression disorder (MDD), maniac, bipolar disorder, schizophrenia, schizoaffective disorder, generalized anxiety disorder (GAD), obsessive–compulsive disorder (OCD), ADHD, conduction disorder, anorexia nervosa, bulimia, and post-trauma stress disorder (PTSD). The last study [10] used K-SADS-E, the epidemiological version of K-SADS (see Table 1).

**Table 1:** Study population characteristics

No.	Study	Country	Total of samples	Age^c^		Diagnostic tool
				ADHD	Control	
1	Aarts et al. [12]	Netherlands	19 ADHD, 77 controls^a^	19.5	27.1	K-SADS based on DSM-IV
2	Jiang et al. [9]	China (Zhejiang)	51 ADHD, 32 controls	8.47	8.5	K-SADS-PL based on DSM-IV
3	Prehn-Kristensen et al. [14]	Germany	14 ADHD^b^, 17 controls^b^	11.9	13.1	K-SADS-PL (Germany translation) based on DSM-IV
4	Wang et al. [10]	China (Taiwan)	30 ADHD, 30 controls	8.4	9.3	K-SADS-E (China version) based on DSM-IV-TR
5	Wan et al. [11]	China (Beijing)	17 ADHD, 17 controls	8 (median)	8 (median)	K-SADS based on DSM-5
6	Szopinska-Tokov et al. [13]	Netherlands	41 ADHD, 15 *subthreshold* ADHD, 47 controls	ADHD = 20,2; *subthreshold* ADHD = 20,2	20.5	K-SADS based on DSM-IV

^a^Control samples consists of 17 healthy participants, 21 healthy siblings of ADHD patients, and 39 self-reported healthy participants diagnosed by Brain Imaging Genetics (BIG)

^b^All the samples are from male participants. ADHD samples consists of 12 participants with combination type and 2 participants with inattention; 6 patients out of 14 have ODD comorbidities; and 10 patients had been consuming ADHD medication for more than a year to treat ADHD (nine of them had agreed to stop the consumption 48 h prior to samples collection)

^c^The data are displayed in mean

#### Microbiota analysis

Four out of six studies used 16s rRNA sequencing, but in different regions. Three studies [9, 10, 12] analyzed region V3–V4 and the other study [13] analyzed region primer 27F-DegS(5′GTTYGATYMTGGTCAG)-338RI-II (5′GCWGCC [T/A] CCCGTAGG [A/T] GT). In addition, the study by Prehn-Kristensen et al. (2018) used DNA amplification on primer 27F-338R and the last study by Wan et al. used shotgun metagenomic sequencing (see Table 2).

**Table 2:** Study population methodology

No.	Study	Genetic analysis	Characteristic of population				
			Medical history	Antibiotic usage	Probiotic/prebiotic usage	ADHD medication	Dietary patterns
1.	Aarts et al. [12]	16S rRNA gene sequencing using Titanium *sequencing chemistry*	No information	No information	No information	No information	No information
		Region: V3–V4					
		Pipeline analysis: QIIME ver 1.2					
		Database: ENA					
2.	Jiang et al. [9]	16s rRNA gene pyrosequencing using Illumina MiSeq *platform *with TruSeqTM DNA Sample Prep Kit	Excluded sample with digestive symptoms, depressive and anxiety symptoms, obesity, atopic diseases (allergic rhinitis, asthma, eczema), and other severe diseases 2 months prior to samples collection	No use of antibiotics 2 months prior to samples collection	No use of probiotic/prebiotic 2 months prior to samples collection	No history of ADHD medication	Excluded vegetarian and non-vegetarian diet (using questionnaire filled by parents)
		Region: V3–V4					
		Pipeline analysis: QIIME					
3.	Prehn-Kristensen et al. [14]	DNA amplification on primer 27F-338R using Illumina Miseq	No information	No information	No information	10 samples of ADHD had been taking ADHD medication for more than a year	The participants took notes on their food intake for fast food, meat/sausage/cold cuts, fruits/vegetables, and yoghurt/dairy products (using 4-point scales)
		Region: V1–V2					
		Pipeline analysis: Mothur					
4.	Wang et al. [10]	16S rRNA gene sequencing	Excluded samples with history of neuropsychiatry disorder or any major diseases	No recent use of antibiotic	No recent use of antibiotic	No history of ADHD medication	Excluded vegetarian diet (using FFQ)
		Region: V3–V4					
5.	Wan. et al. [11]	*Shotgun metagenomic sequencing *using Illumina Novaseq *platform*	Excluded samples with history of respiratory/digestive infection in a month prior to samples	No information	No use of probiotic 1 month prior to samples collection	No information	Excluded vegetarian diet. The participants were asked to maintain their regular dietary patterns for 1
		Pipeline analysis: HUMAnN2 ver 0.11.2					
		Database: *Integrated Gene Catalog *and KEGG	Collection, history of digestive/chronic diseases, BMI < 20 kg/m^2^, and allergic rhinitis/asthma				Week prior to samples collection (using food diary)
6.	Szopinska-Tokov. et al. [13]	16s rRNA gene sequencing	No information	No recent use of antibiotic	No recent use of probiotic	There were history of ADHD medication	No information
		Region: 27F-DegS-338RI-II					
		Pipeline analysis: NG-Tax 16s rRNA					
		Database: SILVA					

#### Diversity analysis (see Table 3)

**Table 3:** Microbial diversity in each study

No.	Study	Alpha diversity index	Alpha diversity	Beta diversity index	Beta diversity
1	Aarts et al. [12]	Shannon, Chao1	No significant differences	–	–
2	Jiang et al. [9]	ACE, Chao1, Shannon, Simpson	No significant differences	*Unweighted and weighted* UniFrac, Bray–Curtis PCoA	Could not be differentiated
3	Prehn-Kristensen et al. [14]	Shannon, Chao1	Decreased (p_Shannon_ = 0.036)	ANOSIM, ADONIS, Betadisper *from the R package vegan v2.4–1*	Differed significantly (p_ANOSIM_ = 0.033; p_ADONIS_ = 0.006; p_betadisper_ = 0.002)
4	Wang et al. [10]	Shannon, Chao1	Shannon index (p = 0.0378) and Chao index (p = 0.0351) were increased significantly in ADHD Simpson index (p = 0.0339) was decreased significantly in ADHD	*Unweighted & weighted* UniFrac, PCoA	No significant differences
5	Wan et al. [11]	Shannon, Chao1, Simpson	No significant differences	–	–
6	Szopinska-Tokov et al. [13]	Shannon–Wiener, Faith’s *phylogenetic diversity*	No significant differences	UniFrac *distance metric*, ADONIS, Betadisper *ver 2.5–2*, PCoA	Differed significantly (10 genera showed nominal difference)

Five studies [9–12, 14] identified α-diversity using Shannon and Chao1 index. The study by Wan et al. also used Simpson index, while Jiang et al. also analyzed the Abundance-based Coverage (ACE) and Simpson index. Only one study [13] used Shannon–Wiener index and Faiths’ phylogenetic diversity.

Two studies [11, 12] did not analyze the β-diversity of their microbiota profiles. Three [9, 10, 13] out of four studies that assessed β-diversity used unweighted and weighted UniFrac and principal coordinates analysis (PCoA). The study by Szopinska-Tokov et al. also assessed ADONIS index and betadisper, while Prehn-Kristensen et al. additionally used ADONIS, ADONIS, and betadisper for their study.

#### ***Findings (see Table ****4**)*

**Table 4:** Microbiota profile found significantly different in ADHD

No.	Study	Gut microbiota profiles
1	Aarts et al. [12]	Phylum: ↑: *Actinobacteria* Order: ↓: *Clostridiales* Family: ↑: *Rikenellaceae, Porphyromonadaceae* Genus: ↑: *Bifidobacterium, Eggerthella*
2	Jiang et al. [9]	Family: ↑: *Peptostreptococcaceae*^a^*, Moraxellaceae*^b^*, Xanthomonadaceae*^b^*, Peptococcaceae*^b^ ↓: *Alcaligenaceae*^a^ Genus: ↓: *Faecalibacterium*^a^*, Dialister*^a^*, Lachnoclostridium*^c^*, Sutterella*^b^
3	Prehn-Kristensen et al. [14]	Family: ↑: *Neisseria, Bacteroidaceae* Genus: ↑: *Neisseria* ↓: *Prevotella* OTU level: ↑: *Bacteroides OTU_7, Bacteroides OTU_577*
4	Wang et al. [10]	Phylum: ↑: *Fusobacteria* Genus: ↑: *Fusobacterium* Species: ↑: *Bacteroides uniformis, Bacteroides ovatus, Sutterella stercoricanis* ↓: *Bacteroides coprocola*
5	Wan et al. [11]	Genus: ↑: *Odoribacter*^d^*, Enterococcus*^b^ ↓: *Faecalibacterium*^d^*, Veillonellaceae*^d^
		Species: ↑: *Bacteroides caccae*^d^*, Odoribacter splanchnicus*^d^*, Paraprevotella xylaniphila*^d^*, Veillonella parvula*^d^*, Roseburia intestinalis*^d^*, Odoribacteraceae*^b^*, Enterococcaceae*^b^ ↓: *Faecalibacterium prausnitzii*^d^*, Lachnospiraceae bacterium*^d^*, Ruminococcus gnavus*^d^*, Ruminococcaceae*^b^
6	Szopinska-Tokov et al. [13]	Genus: ↑: *Clostridiales_g__, Family_XII_AD3011_group, Ruminiclostridium_9, Ruminococcaceae_NK4A214_group, Ruminococcaceae_UCG_003, Ruminococcaceae_UCG_004, Ruminococcaceae_UCG_005, Ruminococcaceae_g_uncultured, Ruminococcus_2, Dialister*^e^ ↓: *Haemophilus, Phascolarctobacterium*^e^

FDR, *false discovery rate*; LEfSe, linear discriminant analysis (LDA) effect size

^a^FDR and LEfSe method

^b^LEfSe method

^c^FDR

^d^Wilcoxon test

^e^ADHD-medicated samples

The total of 49 bacterium taxa were found to be significantly (*p* < 0.05) different between ADHD and healthy controls. The result of each study varied that it was difficult to point out which bacteria taxa differed the most in ADHD. Study from Aarts et al. stated that *Clostridiales* order, *Rikenellaceae* and *Porphyromonadaceae* families, and *Bifidobacterium* and *Eggerthella* genera might be the potential markers for ADHD. The change in *Bifidobacterium* abundance is related to phenylalanine pathway for carbohydrate-deficient transferrin. Genus *Dialister* decreased in the study by Jiang et al. [9], but increased in medicated samples in study by Szopinska-Tokov et al. [13]. Genus *Faecalibacterium* decreased in the study by Jiang et al. [9] and Wan et al. [11], but it is known that the amount of this genus does not correlate with CPRS (Conners Parent Rating Scale) score and the hyperactivity index score [9].

At OTU level, study by Prehn-Kristensen et al. [14] found an increased level of *Bacteroides OTU_7* and *Bacteroides OTU_577.* The abundance of this genus varied between studies. A study by Wang et al. [10] found increased levels of *B. uniformis* and *B. ovatus,* but decreased level of *B. coprocola*. In another study by Wan et al. [11], they found that the *B. caccae* level was increased. The abundance of *B. uniformis, B. ovatus*, and *S. sterrcoricanis* were found to be strongly correlated with the consumption of fat and carbohydrate, and also with ADHD symptoms. In addition, *S. sterrcoricanis* is also associated with specific food intake, including milk, nuts, ferritin, and magnesium.

Family of *Ruminococcaceae* was found to be increased in the study by Szopinska-Tokov et al. [13]. The *Ruminococcaceae_UCG_004* genus especially is associated with inattention score, but not with hyperactivity/impulsivity score. Interestingly, ADHD medication does not affect the abundance of this genus. In this study, it is also found that *Phascolarctobacterium* is decreased in ADHD-medicated samples.

## Discussion

### Main findings

The results are varied between each study and all studies have the distinct taxa findings between ADHD and healthy control groups. Thus, there was minimal consensus that examined which bacterial taxa correlated most to ADHD.

### Risk of bias

#### Differences in sample

Out of six studies, only one study by Wan et al. [11] displayed their age information in median, thus explaining that the distribution of their samples is abnormal. It has been discussed that age is related to the composition of gut microbiota [15]. Other than that, a study by Szopinska-Tokov et al. [11] also included samples from subthreshold ADHD. Study by Prehn-Kristensen et al. [14] is the only study with all-male samples. This actually made the samples more homogenous as gender is also associated with gut microbiota composition. In mice model study, it is found that *Allobaculum, Anaeroplasma*, and *Erwinia* genera are more abundant in male mice, while SMB53 genus from *Clostridiaceae* family and *Dorea, Coprococcus,* and *Ruminococcus* genera are more abundant in female mice [16].

#### Dietary patterns in sample

A study in Korea found that high consumption of fast foods, soft drink, and instant noodles is highly associated with increased K-ARS (Korean version of ADHD Rating Scale) score [17]. Vegetarian diet pattern is known for its effect in increasing the amount of protective species and decreasing the pathologic ones like *Enterobacteriaceae*. In addition, vegetarian diet pattern may reduce inflammation caused by the increased level of *Bacteroides fragilis* and *Clostridium* species, leading to decreased level of intestinal lipocalin-2 and short-chain fatty acids. Lipocalin-2 is a biomarker for inflammation [18]. In ADHD, it is known that IL-6 level is increased because of the gut microbiota dysbiosis [19]. By excluding vegetarian diet pattern, the study may better represent the normal majority of population.

#### Body mass index effect on samples

BMI can affect the composition of gut microbiota, especially those who are overweight or obese. There was an increased ratio of *Firmicutes:Bacteroides* in obese children. In addition, the concentration of *B. vulgatus* decreased while *Lactobacillus *spp*.* concentration increased. *S. staphylococcus* was also known to correlate with energy intake [20]. BMI should be included as a variable in conducting this type of study.

#### ADHD medication effect on samples

There was a decreased α-diversity in ADHD-medicated samples in study by Prehn-Kristensen et al. [14]. In other studies, Szopinska-Tokov et al. [13] found increased level of *Dialister* genus in ADHD-medicated samples, while Jiang et al. [9] found decreased level of this genus as they only included samples who have never been on ADHD medication. The genus *Dialister* is known for its potential in modulating gamma-aminobutyric acid (GABA) neurotransmitter, which may play a role in the pathogenesis of ADHD [21]. We suggest that this genus may be the biomarker for ADHD-medicated patients. There should be a further study just to compare those who have never taken any ADHD medication and those who have taken ADHD medication to find if there are any differences between two groups.

#### Prebiotic/probiotic/antibiotic consumption

Consumption of prebiotic/probiotic/antibiotic will affect the gut microbiota composition. For example, *Bifidobacterium infantis* can decrease pro-inflammatory cytokine in animal model study with irritable bowel syndrome (IBS). Moreover, it is known for its anti-inflammatory effect when consumed with α-linoleic [22]. Other effects of prebiotic/probiotic are widely studied in other disorders such as autism spectrum disorder (ASD), but further study is still warranted to elaborate its benefits in neuropsychiatry disorder [23].

Antibiotics such as penicillins, quinolones, macrolides, sulfonamides, and anti-tuberculosis agents are claimed to induce psychotic behavior. These antibiotics may disrupt the microbiota’s metabolism, therefore affecting the neurotransmitter and SCFA (short-chain fatty acids) which will lead to brain disturbance through GBA [24]. In addition, antibiotics use may also relieve anxiety symptoms and may be helping those with ADHD achieve a calmer mental state. This in turn can possibly affect the levels of gut microbiota in ADHD patients [25]. Furthermore, in an animal model study, an antibiotic regimen of bacitracin, neomycin, and primaricin were given to mice for 7 days and resulted in an increased population of *Lactobacilli* and *Actinobacteria*. This change indicates that antibiotics consumption can alter normal gut microbiota composition.

#### Neuropsychiatric disorder and other diseases effects on samples

Other neuropsychiatric disorders such as depression, schizophrenia, bipolar, Alzheimer’s and Parkinson’s diseases are extensively studied about their correlation with gut microbiota. Several genera are known to induce depression through HPA axis, GABA, SCFAs, immune system and gut barrier [26]. In patient with bipolar disorder, *Actinobacteria* phylum and *Coriobacteria* class were found more abundant compared to healthy individuals [27]. In MDD, the amount *Lactobacillus* (P = 0.067) and *Bifidobacterium* (*p* = 0.012) has been found to be lower [28]; whereas in schizophrenia, it is found that the abundance of *Proteobacteria* was increased. Six genera were found increased (*Succinivibrio, Megasphaera, Collinsella, Clostridium,* Klebsiella, and *Methanobrevibacter*), while three other genera were found decreased (*Blautia, Coprococcus, Roseburia*) [29]. In addition, increased abundance of *Escherichia/Shigella* was found in Alzheimer’s disease and increased *Lactobacillaceae* was found in Parkinson’s disease [30]. Therefore, by excluding other neuropsychiatric disorders, the study may be more accurate. Other diseases, like infection, may affect or be affected by gut microbiota. In human immunodeficiency virus type-1 (HIV-1), for example, it is said that gut microbiota may predict the immune status of the patient. The genera composition in HIV-1 was heavily disturbed and antiretroviral (ARV) medication was implicated with the decrease of *Prevotella* genus [31]. A study in 2018 found that 17 common diseases were associated with at least one microbiota marker (false discovery rate (FDR) < 0.05). Given the example, decreased *Ruminococcaceae* is associated with irritable bowel syndrome [32]. Other than that, type 2 diabetes mellitus (T2DM) was also found to correlate with a smaller amount of phyla *Firmicutes* and *Clostridia* [33].

### Possible link between gut microbiota and ADHD

#### GABA-producer microbiota role in pathogenesis of ADHD

The role of GABA in the pathogenesis of ADHD is still unknown. A study in 2012 found decreased GABA level in ADHD [34]. On the contrary, a study in 2015 stated that adults with ADHD have increased concentration of GABA+, but not in children. This finding also explain that age is positively correlated with GABA+ concentration and ADHD [35]. Another study in 2016 said that there was a negative correlation between GABA level with impulsivity and aggression score [36]. We found several interesting findings in the studies reviewed in this paper. *Bifidobacterium* [12] genus and *Peptostreptococcaceae* [9] family were found increased in ADHD. Furthermore, four species from *Bacteroides *spp. were found increased, while one species was found decreased [10, 11]. Those taxa were known as the GABA producer, especially *Bifidobacterium.* This genus is known to be the most efficient GABA producer [37]. Thus, we contend that GABA may play a role in the pathogenesis of ADHD, but further study is warranted to learn the exact mechanism of this link.

#### Norepinephrine and dopamine role in pathogenesis of ADHD

Though the exact pathogenesis of ADHD is still unclear, if we deduce the mechanism of action of methylphenidate, we can assume that norepinephrine and dopamine play a big role in ADHD. Methylphenidate works by inhibiting the reuptake of dopamine and norepinephrine. D1 dopamine receptor activation (DRD1) regulates NOD-like receptor protein 3 (NLRP3) through cyclic adenosine monophosphate (cAMP), thus destroying NLRP3 in all sites helped by E3 ubiquitin MARCH7. Dopamine and DRD1 may reduce neurotoxin-induced neuroinflammation, lipopolysaccharide (LPS)-induced systemic inflammation, and monosodium urate crystal (MSU)-induced peritoneal inflammation [38]. Norepinephrine, on the other hand, may trigger the growth of protective bacteria like *Escherichia coli* [5]*.* Unfortunately, from all the studies we reviewed, we cannot find a specific gut microbiota that has the ability to modulate or produce dopamine nor norepinephrine. It is still unknown how methylphenidate can affect the composition of gut microbiota of ADHD patients. As stated before, we suggest that the *Dialister* genus may be the biomarker for those with ADHD medication, but further research is needed to identify the mechanism between methylphenidate and gut microbiota.

#### Micronutrient supplementation in ADHD and its effect on gut microbiota

A study in 2019 tried to compare the gut microbiota composition between ADHD children who were given micronutrient treatment and those with placebo. It was found that *Bifidobacterium* genus is decreased in children who were given the micronutrient treatment. The decreased level of *Bifidobacterium* was in line with a decreased in ADHD-IV-RS score [39]. A study by Jiang et al. [9] found an increased abundance of *Bifidobacterium* in ADHD patients. This could hint that micronutrients may restore the balance of *Bifidobacterium*, also that this genus may play a big role in the pathogenesis of ADHD. As explained before, *Bifidobacterium* is one of the many bacteria that can produce GABA. The role of GABA in ADHD still needs to be explored and further study regarding the connection between *Bifidobacterium* and its effect on ADHD symptoms still needs to be analyzed.

#### Effect of pro-inflammatory cytokines modulated by gut microbiota to ADHD pathogenesis

Two studies found a decreased amount of *Faecalibacterium* genus [9, 11]. This genus is known for its anti-inflammatory factors, and a decreased level of this genus may lead to overproduction of pro-inflammatory cytokines [11], thus causing systemic inflammation contributing to ADHD pathogenesis. A study reviewed the possible link between pro-inflammatory cytokines as well as the gene modulating them with ADHD and found increased IL-6 and IL-10 in cytokine protein level [40]. Microglia is activated by pro-inflammatory cytokines, while at the same time also promoting its production. These pro-inflammatory cytokines will lead to a neuroinflammation that may contribute to the pathogenesis of ADHD [41]. Moreover, synaptic plasticity and neurogenesis are also affected by pro-inflammatory cytokines that may alter cognitive processes, such as working memory and reaction time [42]. Further study is still needed to learn more about pro-inflammatory cytokines which are regulated by gut microbiota. This is particularly important as it is a known fact that inflammation process is associated with ADHD pathogenesis.

## Conclusion

Studies comparing the gut microbiota condition between ADHD and healthy individual are still limited. So far there is no common agreement on which bacterial taxa is most relevant to the incidence of ADHD, thus the link between gut microbiota and ADHD remains unclear. Numerous criteria, such as sample size, gender, BMI, dietary pattern, use of prebiotic/probiotic/antibiotic, and history of ADHD medication, should be taken into consideration in conducting this study in the future. In addition, further studies regarding neurotransmitter-modulated gut microbiota are needed, as there are many bacteria whose function remains undiscovered.

## Data Availability

The authors confirm that the data supporting the findings of this study are available within the article.

## Abbreviations

ACE: Abundance-based coverage
ADHD: Attention-deficit hyperactivity disorder
ARV: Antiretroviral
ASD: Autistic spectrum disorder
BMI: Body mass index
cAMP: Cyclic adenosine monophosphate
DRD1: Dopamine receptor D1
DSM: Diagnostic and Statistical Manual of Mental Disorders
FDR: False discovery rate
FFQ: Food Frequency Questionnaire
GABA: Gamma-aminobutyric acid
GAD: Generalized anxiety disorder
GBA: Gut–brain axis
HIV-1: Human immunodeficiency virus type-1
HPA: Hypothalamus pituitary adrenal
IBS: Irritable bowel syndrome
K-ARS: Korean Version of ADHD Rating Scale
K-SADS: Kiddie Schedule for Affective Disorders and Schizophrenia
K-SADS-E: K-SADS epidemiological
K-SADS-PL: K-SADS present and lifetime
LPS: Lipopolysaccharide
MDD: Major depression disorder
MSU: Monosodium urate crystal
OCD: Obsessive compulsive disorder
PCoA: Principal coordinate analyses
PRISMA: Preferred Reporting Items for Systematic Reviews and Meta-Analyses
PTSD: Post-traumatic stress disorder
SCFA: Short-chain fatty acid
T2DM: Type-2 diabetes mellitus
